# The anticonvulsive Phenhydan^**®**^ suppresses extrinsic cell death

**DOI:** 10.1038/s41418-018-0232-2

**Published:** 2018-11-15

**Authors:** Caroline Moerke, Isabel Jaco, Christin Dewitz, Tammo Müller, Annette V. Jacobsen, Jérémie Gautheron, Jürgen Fritsch, Jessica Schmitz, Jan Hinrich Bräsen, Claudia Günther, James M. Murphy, Ulrich Kunzendorf, Pascal Meier, Stefan Krautwald

**Affiliations:** 1 Department of Nephrology and Hypertension, University Hospital Schleswig-Holstein, 24105 Kiel, Germany; 2 Toby Robins Research Centre, Institute of Cancer Research, London, SW3 6JB UK; 3 The Walter and Eliza Hall Institute of Medical Research, Parkville, VIC 3052 Australia; 4 Department of Medical Biology, University of Melbourne, Parkville, VIC 3052 Australia; 5 Université Pierre et Marie Curie, UMR_S 938, Inserm, 75012 Paris, France; 6 Institute for Clinical Microbiology and Hygiene, University of Regensburg, 93053 Regensburg, Germany; 7 Department of Pathology, University of Hannover, 30625 Hannover, Germany; 8 Department of Medicine 1, Friedrich-Alexander-University, 91052 Erlangen, Germany

**Keywords:** Cell biology, Preclinical research

## Abstract

Different forms of regulated cell death-like apoptosis and necroptosis contribute to the pathophysiology of clinical conditions including ischemia-reperfusion injury, myocardial infarction, sepsis, and multiple sclerosis. In particular, the kinase activity of the receptor-interacting serine/threonine protein kinase 1 (RIPK1) is crucial for cell fate in inflammation and cell death. However, despite its involvement in pathological conditions, no pharmacologic inhibitor of RIPK1-mediated cell death is currently in clinical use. Herein, we screened a collection of clinical compounds to assess their ability to modulate RIPK1-mediated cell death. Our small-scale screen identified the anti-epilepsy drug Phenhydan^®^ as a potent inhibitor of death receptor-induced necroptosis and apoptosis. Accordingly, Phenhydan^®^ blocked activation of necrosome formation/activation as well as death receptor-induced NF-κB signaling by influencing the membrane function of cells, such as lipid raft formation, thus exerting an inhibitory effect on pathophysiologic cell death processes. By targeting death receptor signaling, the already FDA-approved Phenhydan^®^ may provide new therapeutic strategies for inflammation-driven diseases caused by aberrant cell death.

## Introduction

Cell death plays a significant role in a variety of human diseases, determining the extent of tissue destruction and, in turn, organ function [1]. The past decade has witnessed significant expansion of our understanding of regulated cell death beyond apoptosis. Programmed necrosis such as necroptosis, which is characterized by plasma membrane rupture and the release of intracellular contents, is an alternate cell death pathway that has been demonstrated to eliminate cells when caspase activity is limited [2]. Furthermore, it has been established that receptor-interacting serine/threonine protein kinase (RIPK) 1, its related kinase RIPK3, and its substrate Mixed lineage kinase domain-like protein (MLKL) constitute the core of the necroptosis machinery, termed the necrosome [3–6]. RIPK1 can be activated by multiple triggers, including signaling by members of the TNF family of cytokines such as TNFα, TLR3 and 4, Interferon receptors, and certain pathogens [7–9]. RIPK1-mediated cell death is best characterized for TNF receptor 1 (TNFR1), and phosphorylation of the necrosome components RIPK1, RIPK3, and MLKL at different sites is a hallmark of the induction of necroptosis [10]. However, cell death is not the only signaling outcome for this receptor; in fact, NF-κB activation is often the dominant response of death receptors.

Increasing evidence from genetic and pharmacologic analyses has shown that RIPK1-induced cell death is a driver of inflammation and contributes to the progression of many cell death-associated conditions, such as myocardial infarction, stroke, sepsis, viral infections, hepatitis, and ischemia-reperfusion injury (IRI), which is an unavoidable consequence after kidney transplantation. Of course, the development of selective therapeutics for all these disorders would be of overriding relevance. However, no pharmacologic inhibitor of RIPK1-mediated cell death is currently in clinical use.

The highly efficient RIPK1 inhibitor 5-(indol-3-ylmethyl)-3-methyl-2-thio-hydantoin, herein termed necrostatin-1 (Nec-1), was identified in 2005 as the first compound to block necroptotic cell death in human and murine cells [11]. It inhibits the kinase activity of RIPK1, and its protective effect against different cell death scenarios was widely interpreted as confirmation of the occurrence of necroptosis. However, because of its short plasma half-life, in vivo studies examining the therapeutic effects of Nec-1 are extremely scarce. Nec-1 was later discovered to be chemically identical to methyl-thiohydantoin-tryptophan, an inhibitor of the potent immunomodulatory enzyme indoleamine 2,3-dioxygenase (IDO) [12], which means that Nec-1 can exert effects on the immune system that are independent of the inhibition of the kinase activity of RIPK1. Nec-1s, an enhanced stable analogue of Nec-1 that does not target IDO, has a much longer half-life in vivo, and is able to interfere with RIPK1-dependent processes, was developed [12]. Necrostatins fail to block necroptosis in the absence of RIPK1 [13], eliminating the therapeutic application of Nec-1s for RIPK1-independent necroptosis. However, a further RIPK1 inhibitor, GSK2982772, has been developed and is currently undergoing Phase-IIa clinical trials for the treatment of inflammatory diseases such as psoriasis and inflammatory bowel disease [14].

Investigators have proposed that RIPK3 represents a more promising target in this scenario of regulated cell death: it is assumed that suppression of the necroptotic pathway at the level of RIPK3 rather than RIPK1 will be more specific and offer greater therapeutic benefits. Indeed, pharmacologic inhibitors that target the kinase function of RIPK3, such as the small-molecule inhibitors GSK’843, GSK’872, GSK’840, dabrafenib, and ponatinib, have been crucial in demonstrating the importance of RIPK3 kinase activity for necroptosis [7, 15, 16]. Nevertheless, high doses of some of these drugs can induce RIPK3-dependent apoptosis, which is consistent with the observation that mice that express D161N RIPK3, a catalytically inactive form of RIPK3, die during embryogenesis [17], likely owing to an inability to suppress RIPK1-directed cell death allosterically [18]. These characteristics limit the potential of these inhibitors as therapeutic drugs for the suppression of regulated cell death. However, mice that express an alternate kinase-dead RIPK3 protein harboring a different point mutation, K51A, develop normally and survive to adulthood [19], which suggests that pharmacologic suppression of RIPK3 kinase activity without inducing apoptosis is feasible.

Necrosulfonamide (NSA), a cell-permeable acrylamide compound that inhibits human, but not murine, MLKL adaptor function via covalent modification of C86, has been successfully used to block necroptosis in vitro [6]. However, NSA contains a reactive moiety and is therefore highly promiscuous. For this reason, no clinical trials have been registered to evaluate its efficacy in an in vivo setting to date.

All of these promising inhibitors of the necrosome components RIPK1, RIPK3, and MLKL lack legal approval, which precludes their application for preclinical RIPK1-mediated diseases and reduces their therapeutic value in patients. Thus, we investigated whether drugs already approved and in clinical use were suited to the inhibition of regulated cell death processes. The underlying rationale is that the pharmacokinetics, side effects and safety profiles of such FDA-approved drugs are already well documented for therapeutic applications. The drugs examined in this study were considered independently of their current clinical applications, and their effects were compared with those of the potent RIPK1 kinase inhibitor Nec-1s. Our data identify the mechanisms of Phenhydan^®^-mediated prevention of death receptor-induced regulated cell death and indicate that an inhibitor of cell death-associated diseases is readily available for clinical application.

## Results

**Fig. 1:**
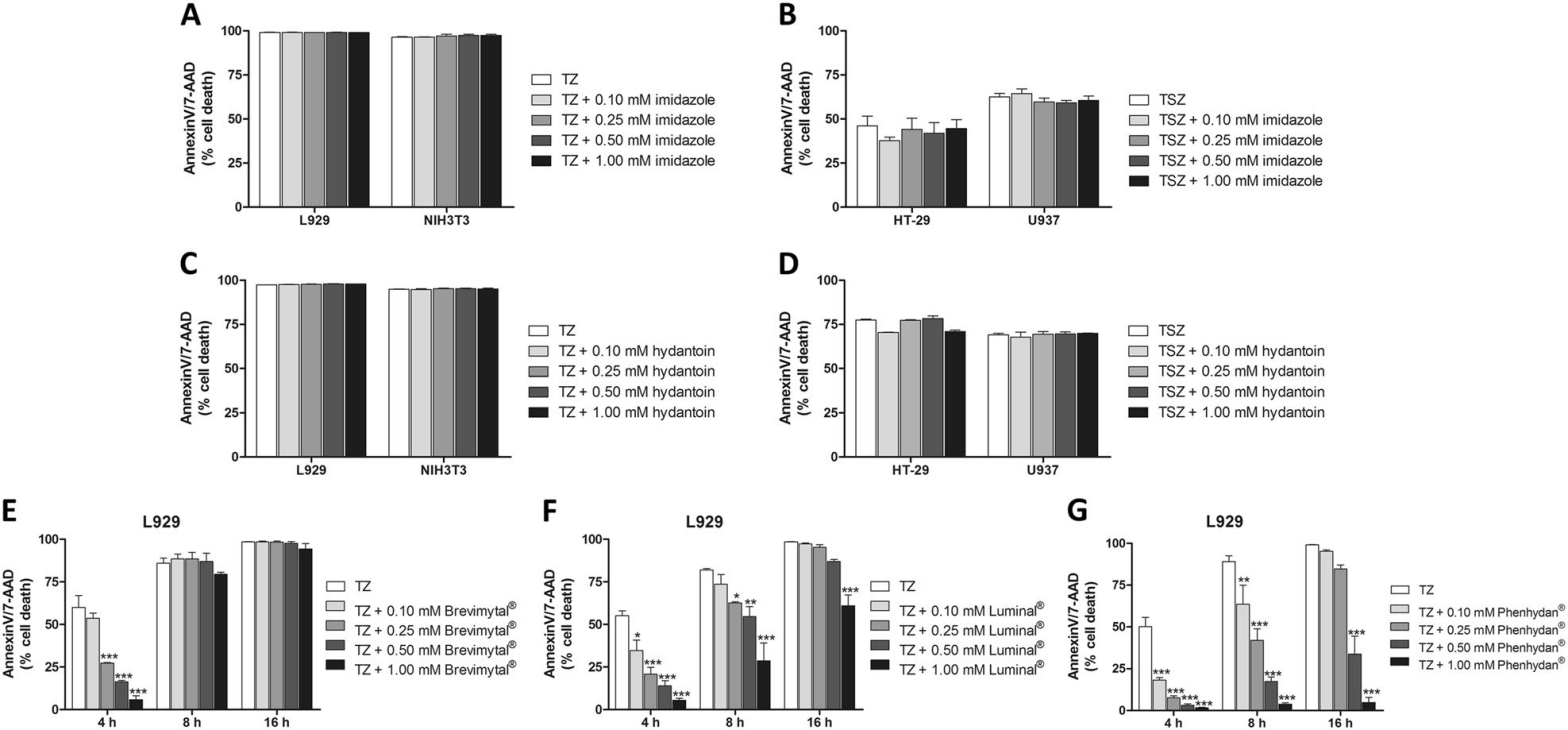
Representative selection of imidazole and hydantoin derivatives based on their inhibitory effect in the course of RIPK1-dependent cell death. **a**, **c** Murine L929 and NIH3T3 cells were stimulated at 37 °C for 16 h with 100 ng/ml TNFα + 25 µM zVAD (TZ) in the absence or presence of different concentrations (as indicated) of unsubstituted imidazole and its derivative hydantoin, respectively. **b**, **d** For the induction of RIPK1-dependent necroptosis, human HT-29 and U937 cells were stimulated at 37 °C for 16 h with 100 ng/ml TNFα + 1 µM SMAC mimetic SM164 + 25 µM zVAD (TSZ) in the absence or presence of the indicated amounts of imidazole and hydantoin. In each case, aromatic compounds were added 30 min before the induction of necroptosis. (E-G) L929 fibroblasts were stimulated at 37 °C for 4, 8, and 16 h with 100 ng/ml TNFα + 25 µM zVAD (TZ) in the absence or presence of Brevimytal^®^ (**e**), Luminal^®^ (**f**), or Phenhydan^®^ (**g**). Each drug was added 30 min before TZ stimulation. Necroptotic cell death was quantified by FACS analysis using 7-amino-actinomycin D and phosphatidylserine accessibility (Annexin V staining) as markers. Graphs show the mean ± SEM; *n* = 4 independent experiments

**Fig. 2:**
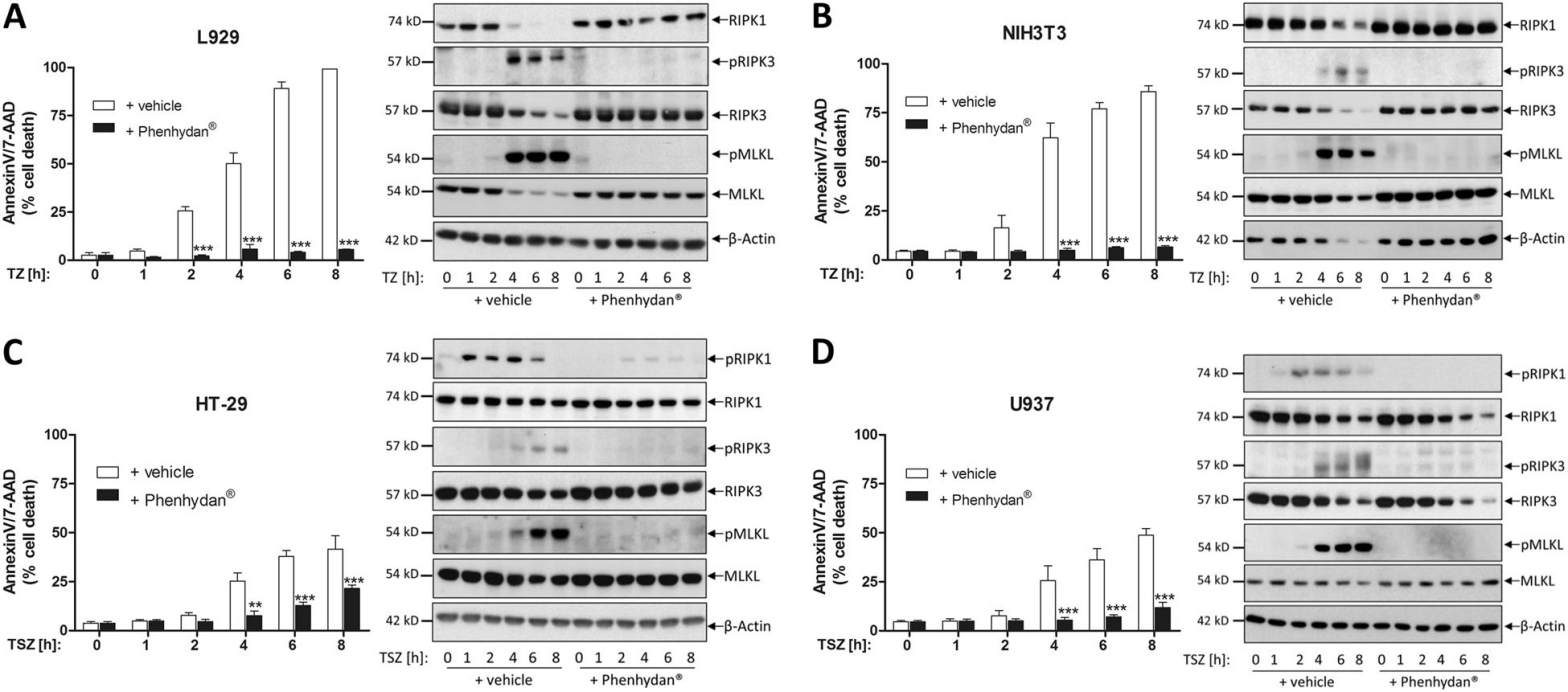
Species-independent confirmation of the inhibitory properties of Phenhydan^®^ in the course of necroptosis. Each probe was split for FACS analysis (left) and corresponding western blotting (right). **a** Murine L929 and murine NIH3T3 cells were stimulated at 37 °C for up to 8 h with 100 ng/ml TNFα + 25 µM zVAD (TZ) in the absence or presence of 1 mM Phenhydan^®^. For the induction of necroptosis, human HT-29 and human U937 cells were stimulated at 37 °C for up to 8 h with 100 ng/ml TNFα + 1 µM SMAC mimetic SM164 + 25 µM zVAD (TSZ) in the absence or presence of 1 mM Phenhydan^®^, wherein the drug Phenhydan^®^ was added 30 min before the induction of necroptosis. Necroptotic cell death was quantified by FACS analysis using 7-amino-actinomycin D and phosphatidylserine accessibility (Annexin V staining) as markers. Graphs show the mean ± SEM; *n* = 4 independent repeats. The whole expression level and activation status (phosphorylation) of the indicated necrosome member receptor-interacting serine/threonine protein kinase 1, receptor-interacting serine/threonine protein kinase 3, and Mixed lineage kinase domain-like protein after necroptosis in the absence or presence of Phenhydan^®^ were analyzed by western blotting, using the indicated specific antibodies. Notably, identical samples (time courses of different cell lines) to those analyzed by FACS were used in this approach. All blots were each re-developed with an antibody against β-actin as a loading control. One representative blot of four independent experiments is shown

To identify an FDA-approved drug that could be used for the treatment of pathophysiologic cell death processes, we selectively screened a small set of clinically approved compounds. In particular, we focused on drugs that are structurally related to Nec-1. To this end, we first tested the unsubstituted heterocyclic aromatic compound imidazole and its derivative hydantoin. We found that these exhibited no inhibitory effect on necroptosis in a range of necroptosis-sensitive cell lines (Fig. 1a–d). Next, we focussed on heterocyclic compounds, which constitute nearly 70% of active pharmaceuticals [20]. Particularly, we tested the effect of barbiturates, which are a class of drugs that produce effects ranging from mild sedation to anesthesia and are used to treat acute convulsive episodes requiring emergency intervention. Intriguingly, barbiturates such as Brevimytal^®^ and Luminal^®^ delayed but did not inhibit TNF-induced and RIPK1-mediated cell death in murine L929 cells (Fig. 1e, f). While Luminal^®^ delayed necroptosis, the more potent structural analogue phenytoin (Phenhydan^®^), which is widely used as antiepileptic, as well as in a broad spectrum of neurologic disorders [21], protected murine L929 cells from TNF-induced cell death in a dose-dependent manner, and for longer time points (Fig. 1g). Phenhydan^®^ not only inhibited RIPK1-mediated cell death in L929 cells but also in murine NIH3T3 as well as human HT-29, and U937 cells, expanding this observation to multiple cell types and species (Fig. 2a–d). In contrast to Phenhydan^®^, a class of related benzodiazepines and benzodiazepine derivatives with an imidazole structure, such as Tavor^®^ and Dormicum^®^, did not exhibit any detectable effect on TNF-mediated cell death (data not shown), suggesting that the observed cell death suppression is specific to Phenhydan^®^ (all substances described and tested in this work, including their structural formulae, are listed in Supplementary Table 1).

Consistent with the notion that Phenhydan^®^ provides protection from RIPK1-mediated cell death, we found that Phenhydan^®^ treatment suppressed phosphorylation and activation of RIPK1 (p-S166), RIPK3 (p-S227 in human and p-T231/S232 in murine cells) and MLKL (p-S358 in human and p-S345 in murine cells) in these cells (Fig. 2a–d). The inhibitory properties of Phenhydan^®^ on TNF-induced cell death were also evident in primary cells such as bone marrow-derived macrophages (BMDCs) and mouse embryonic fibroblasts (MEFs) (Supplementary Fig. 1). The protective effect of Phenhydan^®^ in TNF-induced cell death was found to be as potent as that of the laboratory established but not FDA-approved inhibitors Nec-1s (RIPK1), GSK’872 (RIPK3) and GW806742X (murine MLKL) [22]. Accordingly, we evaluated and illustrated the potent effect of Phenhydan^®^ in TZ-induced cell death and directly compared its effects with the aforementioned commercially available inhibitors (Supplementary Fig. 2).

**Fig. 3:**
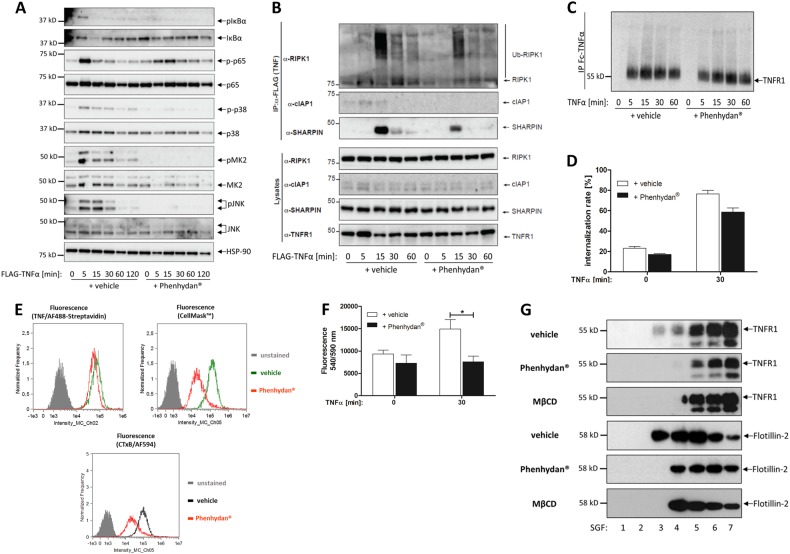
Phenhydan^®^ prohibits canonical NF-κB signaling by influencing the membrane function of cells. Primary MEFs were treated in the absence or presence of 1 mM Phenhydan^®^ with 10 ng/ml TNFα for the indicated durations. Phenhydan^®^ was added 30 min before the addition of TNFα. **a** Western blotting analysis of the cell lysates using the indicated antibodies. **b** TNFα-induced complex-I immunoprecipitation (IP) of primary MEFs treated in the absence or presence of 1 mM Phenhydan^®^ with 1 µg/ml FLAG-tagged TNFα for the indicated durations, followed by FLAG IP and western blotting analysis using the indicated antibodies. **c** Deranged TNF receptor 1 (TNFR1) signaling in the presence of Phenhydan^®^ was ruled out by IP of TNFR1. Cells were treated in the absence or presence of 1 mM Phenhydan^®^ with 100 ng/ml Fc-tagged TNFα for the indicated time points, followed by IP and western blotting analysis using a specific anti-TNFR1 antibody. **d** Quantification of TNFR1 internalization is described in the Materials and Methods section. Labelling of TNFR1 with 100 ng/ml biotinylated-TNFα and 5 µg/ml Alexa Fluor™ 488-conjugated Streptavidin was analyzed over time. The 30 min value ± Phenhydan^®^ is shown representative for imaging flow cytometry. **e** CellMask™ Deep Red plasma membrane staining and subsequent cell analysis revealed the Phenhydan^®^-mediated changes in the lipid bilayer properties, whereas staining in the presence of cholera toxin subunit B (CTxB) confirms that Phenhydan^®^ leads to the disruption of lipid rafts on the cell surface. **f** Acid sphingomyelinase (ASMase) activity assay indicates a distinct decreased activity of ASMase in the presence of Phenhydan^®^. **g** U937 cells were incubated at 37 °C for 30 min with 1 mM Phenhydan^®^ and 10 mM methyl-β-cyclodextrin (MβCD), whereas control cells were vehicle-treated. Membrane lipid rafts and the detergent-soluble fraction were isolated by sucrose gradient centrifugation. From the top of the gradient, 1-ml sucrose gradient fractions were obtained and analyzed by western blotting for the indicated proteins

Since Phenhydan^®^ suppressed auto-phosphorylation of S166 in RIPK1 following TNF treatment, our data suggest that Phenhydan^®^ suppresses early activation of RIPK1 in the TNF receptor signaling complex-I, and that Phenhydan^®^ might suppress TNFR1-signaling in general. Therefore, we evaluated whether Phenhydan^®^ affects TNF-induced NF-κB signaling. Treatment of primary MEFs with TNF in the presence and absence of Phenhydan^®^ demonstrated that sequential phosphorylation of IκBα, p65/RelA, p38 MAPK, MAPK-activated protein kinase-2 (MK2), and JNK after TNF stimulation was largely suppressed by Phenhydan^®^ (Fig. 3a). This strongly suggests that Phenhydan^®^ interferes with TNFR1 signaling. To test whether Phenhydan^®^ affects the recruitment of RIPK1 into TNF receptor signaling complex-I, we stimulated cells with TNF and isolated complex-I. As shown in Fig. 3b, Phenhydan^®^ decisively reduced the recruitment and ubiquitylation of RIPK1 in complex-I. Furthermore, subsequent, time-dependent recruitment of cIAP1 and SHARPIN to the TNFR1 complex upon the addition of TNF was markedly diminished in the presence of Phenhydan^®^ (Fig. 3b). The formation of an abnormal TNFR1 signaling complex in the presence of Phenhydan^®^ may help to explain the inhibitory effect of the drug on RIPK1-mediated cell death. With regard to the mechanism, it is possible that Phenhydan^®^ treatment prevents the autophosphorylation of RIPK1 (a major function of RIP1 kinase in TNF-induced cell death), which enables RIPK1 to recruit RIPK3 and form a functional necrosome [23].

Next, we tested whether Phenhydan^®^ interfered like etanercept (Enbrel^®^) and infliximab (Remicade^®^) with the binding of TNF to TNFR1. Nonetheless, an examination of the internalization kinetics of TNFR1 using labeled TNFα (Fig. 3c) and quantification of this internalization 30 min after ligand binding (Fig. 3d) excluded this possibility. Flow cytometry analysis of TNFR1 on the plasma membrane surface after labelling it with biotinylated TNFα/Alexa Fluor™ 488-conjugated Streptavidin revealed only a slightly lower fluorescence intensity in cells pretreated with Phenhydan^®^ than in vehicle-treated cells (Fig. 3e, upper left). This outcome was in accordance with earlier data on TNF binding and internalization of the ligand/receptor complex, which were not markedly affected by Phenhydan^®^. To explain how Phenhydan^®^ exerts its pharmacologic effects, at least in the context of TNF-mediated signaling, we explored whether this drug induces reorganization of the signaling machinery on the cell surface. The dynamic changes in plasma membranes can be resolved in real time by using lipophilic dyes that are readily incorporated into lipid structures. For our experiments, we used CellMask™ plasma membrane stains, which provide excellent and rapid plasma membrane staining in live cells without any cell-type differences exhibited by Lectins. The dye does not have any biological function but shows strong fluorescence only when intercalated into the hydrophobic core of a biological membrane. The fluorescence spectrum presented in Fig. 3e (upper right) clearly indicates that the membrane structure of the cells, defined as changes in lipid order, is significantly affected in the presence of Phenhydan^®^. To eliminate concerns that Phenhydan^®^ does not mediate a protective effect at the cellular level but only prevents interaction of CellMask™ with the cell surface, we monitored the fluorescence spectrum of CellMask™ before and after the addition of 1 mM Phenhydan^®^ to U937 cells for up to 1 h. Methyl-β-cyclodextrin (MβCD), which is a standard-approved cholesterol-depleting agent, served as an internal control. The data in Supplementary Fig. 3a clearly demonstrate that Phenhydan^®^ does not exert its effect merely by preventing the binding of the plasma membrane dye CellMask™ to the cell surface.

Our results so far are consistent with the notion that lipid rafts are essential for TNFα-mediated activation of TNFR1 but dispensable for the activation of the NF-κB and MAPK pathways [24]. The plasma membrane is composed of discrete lipid microdomains in which membrane molecules are differentially partitioned [25]. Therefore, we used the fluorescein-labeled cholera toxin B subunit (CTxB) to determine whether Phenhydan^®^ engagement affects the distribution of rafts, thereby leading to the alteration of cell death receptor signaling. The binding of CTxB to ganglioside GM1, which is enriched in lipid rafts, is considered as a marker of lipid rafts [26]. As shown in Fig. 3e, the fluorescence intensity of CTxB is markedly declined in the presence of Phenhydan^®^; this supports our hypothesis that the drug leads to increased disruption of lipid rafts. Accordingly, we noticed a distinct decrease in the activity of ASMase in the presence of Phenhydan^®^; this additionally indicates disruption of TNF-signaling downstream of TNFR1 (Fig. 3f).

It has been proposed that TNF receptor-mediated signaling relies on lipid-raft integrity [27]. Upon TNF binding, TNFR1 appears to translocate into these specialized membrane compartments, which are enriched in sphingolipids and cholesterol and serve as sorting platforms for signal transduction proteins. To substantiate our finding that lipid rafts and membrane composition are affected by Phenhydan^®^, we performed sucrose density gradient centrifugation to distinguish between lipid raft and non-lipid raft fractions. To this end, we isolated lipid rafts from whole cells (pretreated ± Phenhydan^®^) and analyzed TNFR1 localization in the separate segregated fractions. Flotillin-2 was used as a lipid raft-associated marker [28]. As shown in Fig. 3g, in the absence of Phenhydan^®^, TNFR1 was abundant in Fractions 3 and 4, representing the detergent-insoluble, glycolipid-enriched microdomains. This outcome is consistent with previous reports that TNFR1 can be recruited to lipid raft-containing fractions [29, 30]. However, in the presence of Phenhydan^®^, little if any TNFR1 was localized to the rafts (Fractions 3 and 4) and instead accumulated in the soluble fraction (Fractions 6 and 7). Treatment with methyl-β-cyclodextrin (MβCD), which is often used to disrupt lipid rafts [31], led to acute depletion of cholesterol from the plasma membrane; thus, the effect of MβCD resembled that of Phenhydan^®^. However, when it was used in an equimolar concentration to Phenhydan^®^ (1 mM), it did not exhibit an inhibitory effect on TNF-mediated cell death (Supplementary Fig. 3b). Nevertheless, it should be mentioned that for effective disorganization of the lipid rafts, as shown before in Fig. 3g, MβCD must be used in a 10-fold higher concentration (10 mM). However, at such a high concentration, it is known [32] that MβCD is toxic to a wide variety of cells already even with a short period of incubation (<1 h). This prevents a direct comparison of the mechanism of MβCD and Phenhydan^®^, with regard to their inhibitory effects in the course of ongoing (>5 h) necroptosis.

Of note, Phenhydan^®^ had no effect on erastin- or RSL3-induced ferroptosis [22], suggesting that the drug does not inhibit any type of regulated cell death (Supplementary Fig. 4a). Furthermore, we found that IL-22-dependent activation of STAT3 in HT-29 cells and GM-CSF-dependent STAT5 sensitivity in U937 cells were unaffected by the presence of Phenhydan^®^ (Supplementary Fig. 4b), indicating that Phenhydan^®^ does not generally block cytokine-mediated signal transduction.

**Fig. 4:**
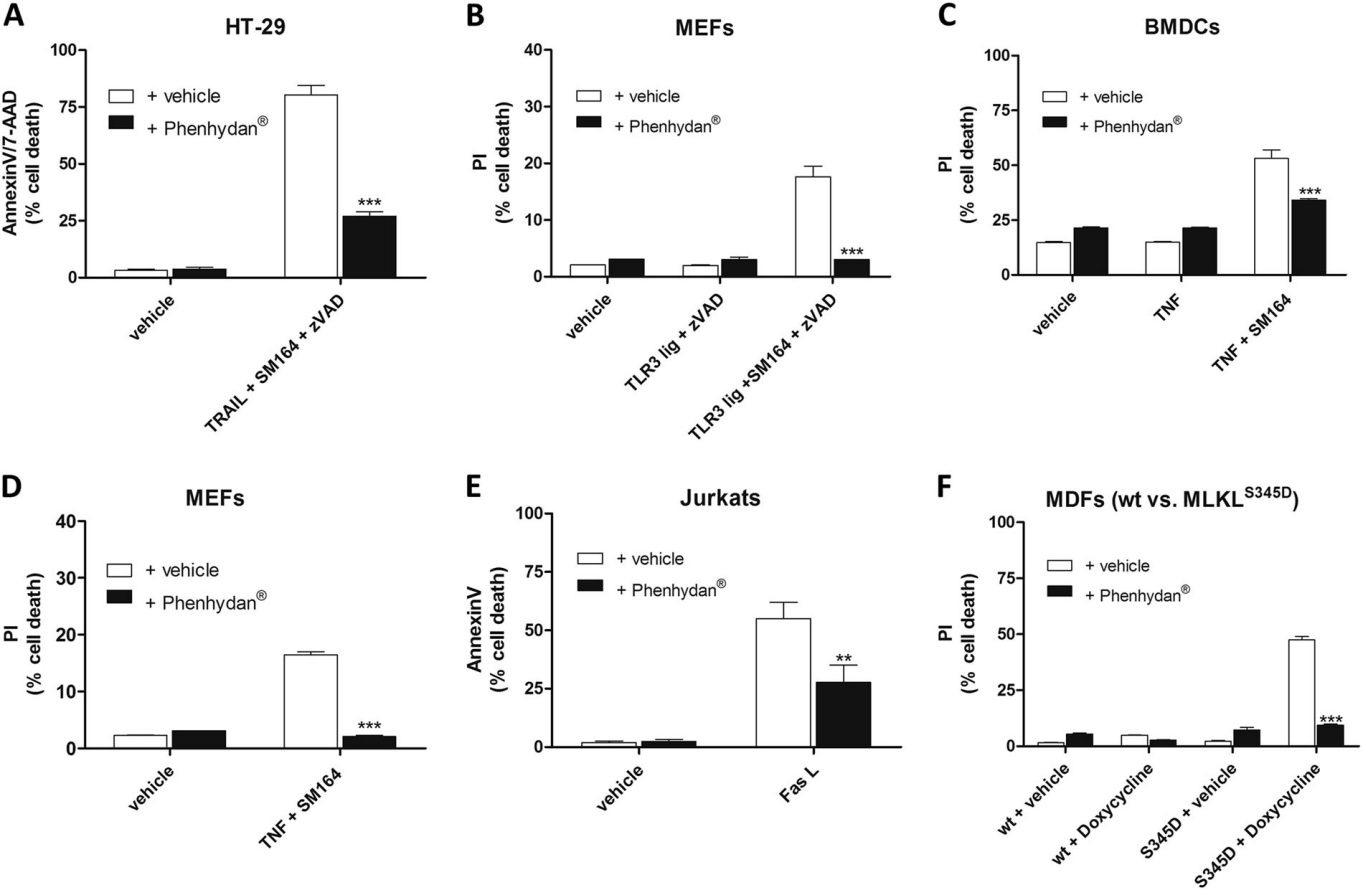
The protective effect of Phenhydan^®^ against regulated cell death is not restricted to TNF-induced necroptosis. **a** HT-29 cells were stimulated at 37 °C for 24 h with 200 ng/ml TRAIL + 1 µM SMAC mimetic SM164 + 25 µM zVAD in the absence or presence of 1 mM Phenhydan^®^. **b** Similarly, primary MEFs were stimulated for 6 h at 37 °C with 10 µg/ml TLR3 ligand + 1 µM SMAC mimetic SM164 + 25 µM zVAD in the absence or presence of 1 mM Phenhydan^®^. Both TRAIL and TLR3 ligand induced necroptotic cell death in a TNF-independent manner. **c**–**e** Phenhydan^®^ was also able to inhibit death receptor-mediated apoptosis. **c** Primary BMDCs and **d** MEFs were stimulated for 3 and 6 h, respectively, at 37 °C with 100 ng/ml TNFα + 1 µM SMAC mimetic SM164 in the absence or presence of 1 mM Phenhydan^®^. **e** Furthermore, human Jurkat cells were stimulated for 4 h at 37 °C with 100 ng/ml FasL. **f** Wild-type or mutated (S345D Mixed lineage kinase domain-like protein) mouse dermal fibroblasts (MDFs) were induced at 37 °C with 0.5 µg/ml doxycycline for 7 h, before cells were harvested and permeabilized in Frackelton buffer. As indicated, 1 mM Phenhydan^®^ was added 30 min before induction. The cell death that occurred in **a**–**f** was quantified by FACS analysis using the indicated markers. Graphs show the mean ± SEM; *n* = 2–3 independent experiments

Since Phenhydan^®^ suppresses TNFR1 signaling, we next tested whether it inhibits other pathogen recognition- or death receptor-initiated processes. To this end, we treated cells with FasL, TRAIL, and TLR agonists, which can induce apoptosis and necroptosis, depending on stimuli. As shown in Fig. 4a–e, we found that Phenhydan^®^ suppressed FasL-, TRAIL-, and TLR3-agonist induced cell death, which strongly suggests that Phenhydan^®^ interferes with death receptor signal transduction, most likely at an early stage such as their integration into lipid rafts. TLR3 is not a death receptor but is localized to and signals from acidified compartments of the endolysosomal pathway, and it mainly plays a role in the detection of nucleic acids of pathogens [33]. Nevertheless, inhibition or elimination of caspase-8 during stimulation of TLR3 results in RIP3 kinase-dependent programmed necrosis that occurs through TIR domain-containing adapter-inducing interferon-β (TRIF) [34]. TRIF, RIPK3, and MLKL promote cell death via a pathway that is analogous to the RIPK1–RIPK3–MLKL axis [7]. This mechanism may explain the protective effect of Phenhydan^®^ in TLR3-initiated cell death. Therefore, we assessed whether Phenhydan^®^-mediated changes in the plasma membrane also affected the ability of MLKL to form pores, regardless the fact that the precise mechanism of this permeabilization has not yet been clarified so far [35–37]. To this end, we made use of a c*o*nstitutively active form of MLKL (phosphomimetic S345D). Doxycycline-inducible expression of this MLKL mutant was reported to cause necroptosis independently of upstream signals [38]. Intriguingly, while induced expression of MLKL^S345D^ readily killed cells, in the presence of Phenhydan^®^ MLKL^S345D^ was significantly less potent in killing cells (Fig. 4f). This suggests that Phenhydan^®^-mediated changes of the lipid bilayer affect the ability of MLKL to form membrane apertures. At the membrane, it may either influence bilayer properties/structures or access target membrane proteins such as voltage-gated sodium channels to exert its pharmacologic action. The possibility that the mere presence of Phenhydan^®^ prevents or significantly reduces the doxycycline-induced expression of the constitutively active MLKL^S345D^, which may lead to an incorrect attribution of protection from necroptosis, was ruled out by direct comparison between MDFs isolated from MLKL-ko animals, MDFs from MLKL-ko animals in which wild-type MLKL was reconstituted, and the constitutively active MLKL mutant S345D. At 7 h after the addition of doxycycline, all inducible cells showed virtually identical protein expression of MLKL regardless of the presence or absence of Phenhydan^®^ (Supplementary Fig. 5).

**Fig. 5:**
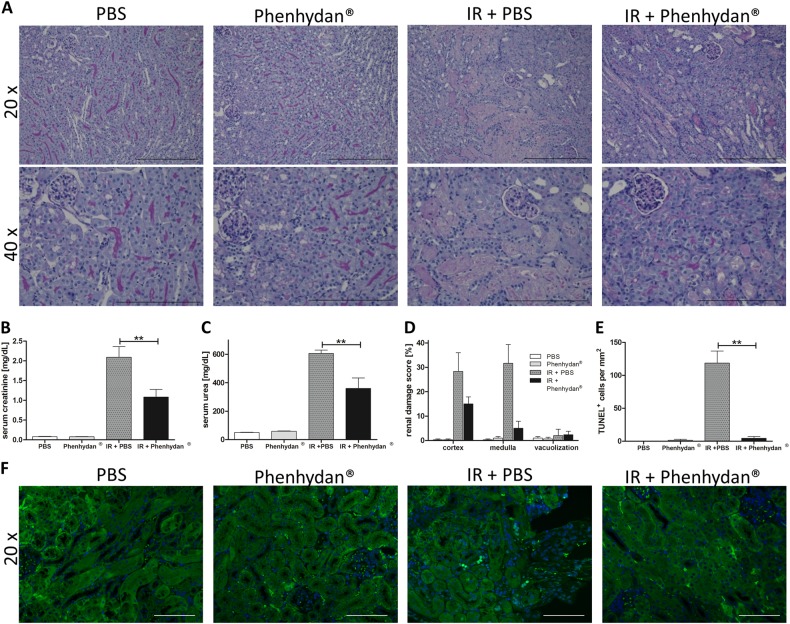
Phenhydan^®^ provides protection from ischemia-reperfusion damage. The significant therapeutic effect of Phenhydan^®^ was evident under exceptionally severe test conditions. For establishing a renal ischemia-reperfusion injury model, all the mice (*n* = 8 per group) underwent 47 min of bilateral renal pedicle clamping followed by 48 h of reperfusion. In the verum group, Phenhydan^®^ was added at a final concentration of 1 mM to the drinking water (renewed every day) 7 days before the onset of ischemia and until the end of the reperfusion phase. **a** Periodic acid-Schiff-stained histomicrographs of the kidneys exhibited diminished total organ damage in the Phenhydan^®^-treated mice compared with the vehicle-treated animals (scale bar = 300 µm [20×] and 200 µm [40×], respectively). Accordingly, we observed that the vehicle-treated mice had significantly lower plasma levels of serum creatinine (**b**) and urea (**c**) than the Phenhydan^®^-treated animals. **d** The protective effect of Phenhydan^®^ in this model was confirmed by quantification of the renal damage. **e**, **f** To illustrate the protective effect of Phenhydan^®^ at the cellular level, a TUNEL fluorescence assay was performed to detect (**f**) and quantify (**e**) cells undergoing regulated necrotic death (in green) in this clinically relevant in vivo model. DAPI was used for nuclear counterstaining (blue). Scale bar = 100 µM

The main purpose of our study was to identify an FDA-approved drug that, independent of its clinical application, possesses an inhibitory effect on pathophysiologic cell death, which might be suitable for emergency use. Since many inflammatory pathologies are driven by aberrant TNF-induced cell death, we tested whether Phenhydan^®^ could be used in vivo to suppress death receptor-induced cell death. To this end, we focused on clinically relevant in vivo models associated with pathophysiologic death receptor-induced cell death, such as renal IRI and immune-mediated liver injury [39, 40]. Renal ischemia causes as well as frequently exacerbates already existing acute kidney disease (AKI) and chronic kidney disease [41]. For the mouse mode of IRI, animals underwent 47 min of bilateral renal pedicle clamping followed by 48 h of reperfusion. As described previously, such long-lasting ischemia is lethal in untreated wild-type mice 72 h after reperfusion [39]. In this setting, functional markers for the loss of kidney function (elevated serum concentrations of creatinine and urea) were significantly reduced 48 h after reperfusion in the presence of Phenhydan^®^ (Fig. 5b, c). This indicates the effectiveness and therapeutic potential of Phenhydan^®^ for the treatment of complex diseases driven by death receptor-induced cell death. Our conclusion is supported by representative periodic acid-Schiff-stained histomicrographs of the kidneys (Fig. 5a) and corresponding quantification of the injury based on the renal damage scores (Fig. 5d). The well-established TUNEL fluorescence assay, which is used to detect (Fig. 5f) and estimate the number of cells undergoing regulated necrotic death (Fig. 5e), confirmed at the cellular level the protective effect of Phenhydan^®^ in this clinically relevant in vivo model.

**Fig. 6:**
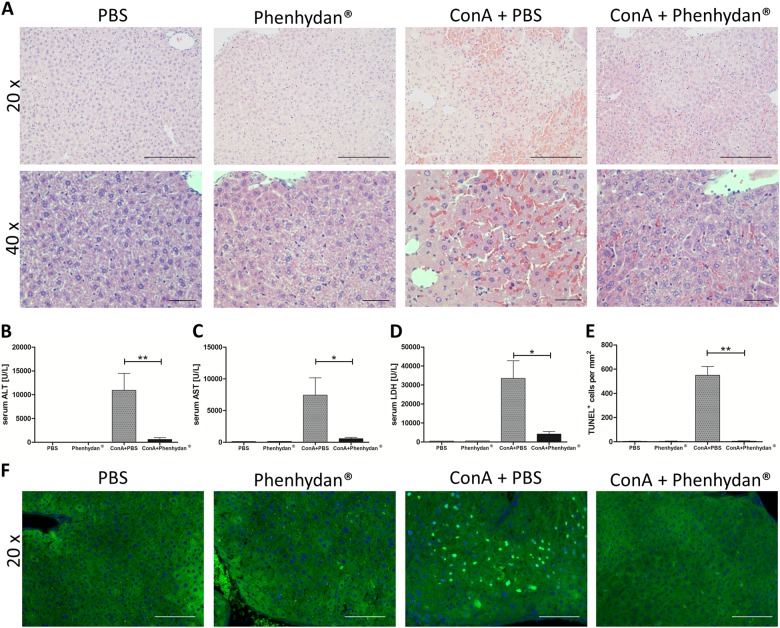
Phenhydan^®^ has protective effects in an in vivo model of inflammation-dependent hepatitis. The beneficial effects of Phenhydan^®^ in diseases involving pathophysiologic necroptosis were confirmed in a murine model of inflammation-dependent hepatitis. Mice (*n* = 10 per group) were subjected to ConA or saline treatment as described in the Materials and Methods section and analyzed 7 h after administration. **a** Immunohistochemical analysis revealed distinctly reduced liver tissue necrosis and hepatocyte death in the Phenhydan^®^-treated mice compared with the vehicle-treated animals (scale bar = 200 µm [20×] and 50 µm [40×], respectively). **b**–**d** Compared with the control group, the Phenhydan^®^-treated animals had considerably reduced plasma alanine aminotransferase (ALT) and aspartate aminotransferase (AST) concentrations as well as considerably reduced lactate dehydrogenase (LDH) activity. **e**, **f** To visualize the protective effect of Phenhydan^®^, we performed a TUNEL fluorescence assay to detect (**f**) and quantify (**e**) cells undergoing regulated necrotic death (in green) in this model of liver injury. DAPI was used for nuclear counterstaining (blue). Scale bar = 100 µM

To further corroborate the use of Phenhydan^®^ as a potential therapeutic agent for the treatment of diseases involving pathophysiologic cell death by death receptors, we use a murine model of inflammation-dependent hepatitis. Hepatitis was induced by intravenous injection of the Lectin ConA, which causes symptoms that resemble immune-mediated hepatitis in humans [42]. In this model MLKL seems to act as a central player because *Mlkl*-knockout animals are protected from ConA injury and experimental hepatitis [40]. While treatment with ConA resulted in liver damage and hepatitis, co-treatment with Phenhydan^®^ greatly diminished plasma alanine aminotransferase (ALT) and aspartate aminotransferase (AST) concentrations, as well as considerably reduced lactate dehydrogenase (LDH) activity (Fig. 6b–d). Moreover, immunohistochemical analysis of these mice revealed significantly reduced liver tissue cell death following Phenhydan^®^ treatment (Fig. 6a). Just as in the IRI model, a TUNEL fluorescence assay was used to monitor (Fig. 6f) and evaluate the number of cells undergoing regulated cell death (Fig. 6e) in this clinically relevant in vivo model. The extent of cell death induced by ConA, and especially the impressive protective effect of Phenhydan^®^, is illustrated in Fig. 6e, f.

## Discussion

Receptor-interacting serine/threonine protein kinase 1 (RIPK1) has been shown to be a critical driver of various pathways downstream of the death receptors TNFR1, FasL, and TRAIL, as well as toll-like receptors. Hence, blocking these pathways has the potential to result in a broad therapeutic benefit for multiple diseases such as ischemia-reperfusion injury, myocardial infarction, sepsis, and multiple sclerosis. Since TNFα is involved in mediating a plethora of human diseases, the inhibition of RIPK1 may benefit patients beyond the indications that are currently being tested. Above all, GlaxoSmithKline (GSK) and Denali Therapeutics have identified and optimized a set of RIPK1 inhibitors and several human clinical trials as treatments of inflammatory and neurodegenerative diseases from ulcerative colitis and rheumatoid arthritis to amyotrophic lateral sclerosis (ALS) and Alzheimer’s disease (AD) have been initiated [43]. Nevertheless, none of these exciting compounds reached Phase-III clinical studies today and accordingly, no pharmacologic inhibitor of RIPK1-mediated cell death is currently in clinical use.

In the present study, we screened a selection of FDA-approved drugs with structures related to the RIPK1 inhibitor necrostatin-1 and identified the anti-epileptic drug Phenhydan^®^ in multiple cell types as a potent inhibitor of both pro-inflammatory NF-κB signaling and aberrant cell death. Furthermore, we were able to show that Phenhydan^®^ elicits these effects by influencing the membrane structure and function of cells. The initial formation of TNF/TNFR complexes is followed by secondary multimerization into supramolecular clusters, and it is known that efficient signal initiation requires the formation of larger ligand/receptor complexes [44]. It has been shown previously that besides TNF/TNFR complex, a considerable fraction of Fas is constitutively partitioned into cholesterol- and sphingolipid-rich lipid rafts within the plasma membrane prior to ligation at a level that is not changed after Fas engagement. However, Fas ligation might trigger compositional changes in lipid rafts, suggesting that rafts represent the membrane site from which, upon engagement by its ligand, Fas initiates its signaling cascade [45]. Similarly, ligation of TRAILR1/2 localized to lipid rafts induces pro-cell death signaling, whereas TRAIL receptors that are not associated with lipid rafts mediate the activation of NF-κB [46]. Interestingly, it was reported that drugs, which induced the localization of TRAILRs to lipid rafts, resulted in enhanced TRAIL-mediated cell death [47]. According to our present data, Phenhydan^®^ has the opposite effect. This is consistent with the protective effect of the drug in the course of regulated cell death. Changes in lipid bilayer properties and lipid raft formation at an early stage alter membrane function, providing an indirect way for this drug to interfere with death receptor signal transduction, which goes beyond a simple modulation of necrosome formation/activation and merely inhibition of necroptotic cell death. The heterogeneity of the lipid composition of different cell lines is well known. Further, the number of physically distinct lipid raft domains in living cells is likely to be enormous [48]. Therefore, the clear separation of detergent-insoluble fractions from soluble ones is not often possible, and the degree of separation differs across cell lines. Since amphiphilic pharmacological compounds such as Phenhydan^®^ can be adsorbed at the bilayer interface and alter lipid bilayer properties [49], it is likely that Phenhydan^®^ acts as a modulator of death receptor signaling by affecting the lipid bilayer properties of the plasma membrane, thus modifying the behavior of a range of membrane-bound proteins, including TNFR1.

Thus, changes in lipid bilayer properties, such as their ability to form lipid rafts or allow MLKL pore formation, could ultimately alter death receptor-mediated apoptosis and necroptosis. Phenhydan^®^, therefore, might influence death receptor signaling in lipid rafts, and conditional redistribution of TNFR1 in the plasma membrane is only one of the many possible mechanisms by which this drug regulates the efficiency of TNF signaling. At the molecular level, our statement is supported by the experiment shown in Fig. 3f. Determination of ASMase activity in the presence or absence of Phenhydan^®^ was performed only in the presence of TNF but without the addition of the pan-caspase inhibitor zVAD. Upon binding of TNF to its corresponding receptor, internalization of this complex occurs immediately, and it was shown previously that this event causes rapid endosome formation [50]. The receptor-containing endosomes fuse with lysosomes, resulting in the formation of multivesicular bodies and bringing lysosomal ASMase into close contact with caspases, which cleave the 72 kDa pro-form of ASMase into a 57 kDa ASMase fragment and thereby activate the enzyme. Our data indicate that ASMase activation after TNF/TNFR1 internalization is significantly inhibited in the presence of Phenhydan^®^.

Moreover, by using two mouse models of death receptor-driven pathology our data clearly demonstrate that Phenhydan^®^ treatment suppresses death receptor-induced cell death also in vivo. On the one hand, we found that the anticonvulsive drug Phenhydan^®^ attenuated functional markers of acute kidney injury (AKI) and histological damage, indicating the effectiveness and therapeutic potential of Phenhydan^®^ for the treatment of complex processes driven by death receptor-induced cell death like ischemia-reperfusion injury (IRI). In this context should be mentioned that AKI is associated with a high morbidity rate and is potentially lethal, and is therefore an emergency medical problem. Our limited understanding of the complex cell death mechanism in the process of AKI impedes the development of desirable therapeutics. To date, there is no effective therapy for AKI in clinical routine. Furthermore, we found that Phenhydan^®^ ameliorates inflammation-induced programmed hepatocyte cell death, suggesting that clinical application of the drug in patients with steatosis and primary biliary cirrhosis is feasible with an inhibitor that is already available.

Given our findings, we suggest to repurpose Phenhydan^®^ in settings where aberrant death receptor-mediated cell death might contribute to human inflammation driven pathologies, such as myocardial infarction, stroke, and organ/graft IRI. The well-defined safety profile of Phenhydan^®^ which is currently used in the clinic as anticonvulsant recommends it for Phase-III clinical trials in which the utility of the drug for the treatment of the aforementioned cell death-associated diseases should be verified. The application for a multicenter, randomized study using Phenhydan^®^, in which the objective is to reduce infarct volume during stent-retriever thrombectomy in acute ischemic stroke by inhibiting regulated cell death, has recently been submitted.

## Materials and methods

### Cell lines

L929, NIH3T3, HT-29, U937, and Jurkat cells were obtained from the American Type Culture Collection (Manassas, VA, USA). The isolation and immortalization of mouse dermal fibroblasts (MDFs) from the dermis of *Mlkl*-knockout and congenic wild-type mice have been described previously [51]. The MDF cell lines, L929, NIH3T3, and HT-29 were cultured in DMEM (Gibco^®^; Thermo Fisher Scientific) supplemented with 10% (vol/vol) FCS (PAN-Biotech GmbH, Aidenbach, Germany), 100 U/ml penicillin, and 100 μg/ml streptomycin (Merck Millipore GmbH, Darmstadt, Germany). Jurkat cells were cultured in RPMI 1640 medium (Gibco^®^; Thermo Fisher Scientific) supplemented with 5% (vol/vol) FCS, 100 U/ml penicillin, and 100 μg/ml streptomycin. U937 cells were cultured in RPMI 1640 medium supplemented with 10% (vol/vol) FCS, 100U/ml penicillin, 100 μg/ml streptomycin, 1 mM sodium pyruvate, and 0.25% D-( + )-glucose (Sigma-Aldrich Chemie GmbH, Taufkirchen, Germany). All cell lines were cultured in a humidified 5% CO_2_ atmosphere.

### Primary cells

Primary MEFs were generated from E13.5 embryos. After removing the placenta, yolk sac, head, and dark red organs, the embryos were finely minced and digested for 20 min in 0.25% trypsin. A single-cell suspension was then obtained by pipetting the digested embryos up and down. To generate bone marrow-derived macrophages, bone marrow cells from the tibias and femurs of 2-month-old mice were seeded in non-coated Petri dishes and cultured for 6 days in DMEM supplemented with 10% (vol/vol) FCS and 20% (vol/vol) L929 mouse fibroblast-conditioned medium. Primary cells were cultured in a humidified 5% CO_2_ atmosphere.

### Reagents and antibodies

Recombinant purified TNFα, the Annexin V-FITC antibody, and the 7-amino-actinomycin D antibody were purchased from BioLegend (London, UK). Recombinant untagged TRAIL, TLR3 ligand, FLAG-tagged TNFα, GM-CSF, IL-22, anti-cIAP1, and anti-Heat shock protein 90 were obtained from Enzo Life Sciences GmbH (Lörrach, Germany). Fc-tagged human TNFα was manufactured by SinoBiological, the bivalent SMAC mimetic SM164 by AdooQ Biosciences, and RSL3 by Selleckchem (all purchased from Hölzel Diagnostika Handels GmbH, Cologne, Germany). Biotinylated TNFα was purchased from Invitrogen (Carlsbad, CA, USA). Alexa Fluor™ 488-conjugated Streptavidin, CellMask™, and cholera toxin subunit B (CTxB) Alexa Fluor™ 594 conjugate were purchased from Thermo Fisher Scientific (Darmstadt, Germany). The pan-caspase inhibitor zVAD-fmk was purchased from Bachem (Weil am Rhein, Germany). ConA was obtained from Merck Millipore. α-human Fas (clone 7C11) was purchased from Immunotech (Marseille, France). PR619, the monoclonal α-FLAG M2 antibody, sucrose, MβCD, imidazole, hydantoin, and propidium iodide (PI) solution were purchased from Sigma-Aldrich Chemie GmbH. Drugs tested in this work were obtained from followed companies: Tavor^®^ (Pfizer Pharma GmbH, Berlin, Germany), Dormicum^®^ (Roche Pharma AG, Reinach, Switzerland), Brevimytal^®^ (Hikma Pharma GmbH, Martinsried, Germany), Luminal^®^ and Phenhydan^®^ (both from Desitin Arzneimittel GmbH, Hamburg, Germany). The anti-ubiquitin antibody was obtained from Dako (Biozol Diagnostica Vertrieb GmbH, Eching, Germany) and the α-SHARPIN antibody was purchased from Proteintech (Manchester, UK). Erastin, GSK’872 and the anti-MLKL antibody (clone 3H1) were obtained from Calbiochem^®^ (Merck Millipore). The murine MLKL inhibitor GW806742X was purchased from Biomol (Hamburg, Germany). Ferrostatin-1 was purchased from Xcess Biosciences Inc. (San Diego, CA, USA). The anti-human as well as the anti-mouse monoclonal phospho-MLKL antibodies, the anti-cholera toxin subunit B (CTxB) antibody, and the anti-TNFR1 antibody were obtained from Abcam (Berlin, Germany). The anti-RIPK3 antibody (human- and mouse-specific) was obtained from ProSci Incorporated (Hölzel Diagnostika Handels GmbH). Anti-RIPK1 (human- and mouse-specific), anti-human and anti-murine phospho-RIPK1, anti-murine phospho-RIPK3, anti-JNK, anti-phospho-JNK, anti-IκBα, anti-phospho-IκBα, anti-MK2, anti-phospho-MK2, anti-p38, anti-phospho-p38, anti-p65, anti-phospho-p65, anti-phospho-STAT3/5, anti-Flotillin-2, and the β-actin antibody were purchased from Cell Signaling Technology Europe BV (Frankfurt, Germany).

### Assessment of cell death in vitro

Phosphatidylserine exposure to the outer cell membrane of apoptotic cells or inner plasma membrane of necrotic cells and incorporation of 7-amino-actinomycin D into necrotic cells was quantified by FACS analysis. Stainings were performed according to the manufacturer’s instructions (BioLegend). Fluorescence was analyzed using an FC-500 flow cytometer (Beckman Coulter GmbH, Krefeld, Germany).

### Analysis of cell death by western blotting

For immunoblotting, cells were lysed in ice-cold 10 mM Tris-HCl (pH 7.5), 50 mM NaCl, 1% Triton X-100, 30 mM Na_4_P_2_O_7_, 50 mM NaF, 100 μM Na_3_VO_4_, 2 μM ZnCl_2_, and 1 mM C_7_H_7_FO_2_S (PMSF) (modified Frackelton buffer). Insoluble material was removed by centrifugation (14,000 × *g*, 10 min, 4 °C) and protein concentration was determined using a commercial Bradford assay kit according to the manufacturer’s instructions (Bio-Rad Laboratories GmbH, Munich, Germany). Equal amounts of protein (20 μg per lane) were resolved by reducing SDS/PAGE and transferred to a polyvinylidene fluoride (PVDF) membrane (GE Healthcare Life Sciences, Freiburg, Germany). Western blots were performed using the specific primary antibodies mentioned above and corresponding secondary horseradish peroxidase-linked polyclonal antibodies obtained from Abcam (Berlin, Germany). Immune complexes were visualized by enhanced chemiluminescence (ECL).

### Mice

Mice used in this study were of the C57BL/6 background and were purchased from Janvier Labs (Saint Berthevin Cedex, France). All mice were carefully matched for age, sex, and weight. For our analyses, the animals used were male mice at an age of 8 weeks. All mice were kept on a standard diet and a 12-h day-night rhythm. All in vivo experiments were performed according to the *Protection of Animals Act* with the approval of German and French local authorities.

### Ischemia-reperfusion injury

Induction of murine kidney IRI was performed via a midline abdominal incision and bilateral renal pedicle clamping for 47 min using microaneurysm clamps (Aesculap, Inc., Center Valley, PA USA). Throughout the surgical procedure, the body temperature of the mice was maintained at between 36 °C and 37 °C by continuous monitoring using a temperature-controlled self-regulated heating system (Fine Science Tools GmbH, Heidelberg, Germany). After removal of the clamps, reperfusion of the kidneys was confirmed visually. The abdomen was closed in two layers using standard 6-0 sutures. To maintain fluid balance, all of the mice were supplemented with 1 ml of prewarmed PBS administered intraperitoneally directly after surgery. The mice were sacrificed 48 h after reperfusion. Serum creatinine and urea values were measured in the Central laboratory of the University Hospital Schleswig-Holstein, Kiel (Germany). IRI experiments were performed in a double-blinded manner. Where indicated, Phenhydan^®^ was added at a final concentration of 1 mM to the drinking water (renewed every day) of the mice 7 days before the onset of ischemia and until the end of the reperfusion phase.

### In vivo model of liver injury

C57BL/6 mice were subjected to ConA or saline treatment and sacrificed 7 h later. ConA was administered intravenously at a concentration of 15 mg/kg body weight. Where indicated, a singular injection of Phenhydan^®^ was administered intraperitoneally at a concentration of 34 mg/kg body weight (total volume per mouse was 200 µl) 30 min before ConA administration. Appropriate amount of PBS (200 µl per mouse) was applied as vehicle control. The plasma concentrations of alanine aminotransferase, aspartate aminotransferase, and lactate dehydrogenase were measured in the Central laboratory of the Université Pierre et Marie Curie, Paris (France) as well as in the Central laboratory of the University Hospital Schleswig-Holstein, Kiel (Germany).

### Histologic, immunohistochemical, and morphologic assessments

Kidney and liver biopsies were fixed in 4% neutral-buffered formaldehyde and embedded in paraffin. The 3-μm sections produced were dewaxed, rehydrated, and subjected to periodic acid-Schiff staining (kidney) and haematoxylin and eosin (liver) according to routine protocols. Stains were evaluated blinded by an experienced pathologist. Sections were evaluated using an U-DO3 microscope (Olympus Corp., Tokyo, Japan). Representative photomicrographs were taken using a Zeiss system (an Axioplan microscope with an MRT digital camera and Axiovision Software; Carl Zeiss AG, Oberkochen, Germany).

To analyze cell death of the tissue sections, the TdT-mediated dUTP nick end labeling (TUNEL) assay was performed using the fluorescence detection kit according to the manufacturer’s instructions (Promega, Mannheim, Germany). Briefly, tissue sections were dewaxed, rehydrated, fixed in 4% paraformaldehyde and permeabilized with Proteinase K for 10 min at RT. Following this, the sections were equilibrated with the provided buffer for 10 min and labeled with the TdT reaction mix for 60 min at 37 °C in a humidified dark environment. To stop the labeling reactions, sections were incubated with the provided stopping buffer for 15 min at RT in the dark. The sections were then washed with PBS for 5 min. Finally, the sections were mounted with fluorescence-mounting media (Dako, Glostrup, Denmark) containing 4′,6-Diamidin-2-phenylindol (DAPI) for cell nuclei counterstaining. Fluorescence micrographs were taken using Zeiss Axio Imager Z1 fluorescence microscope (Carl Zeiss AG, Oberkochen, Germany) at magnifications of 20× and 40× magnification using a standard fluorescein filter set to view the green fluorescence at 520 nm, and blue fluorescence of DAPI at 380 nm. Quantification of TUNEL-positive cells was performed manually by two blinded observers and reproduced in triplicate by each of them.

### Complex-I purification

MEFs were seeded in 15-cm dishes and treated as indicated with 3x FLAG-TNFα (5 mg/ml). The medium was removed and the plates were washed with ice-cold PBS. Then, the plates were frozen at−80 °C. The plates were later thawed on ice and the cells were lysed in 1% Triton X-100 lysis buffer (30 mM Tris-HCl [pH 7.4], 120 mM NaCl, 2 mM EDTA, 2 mM KCl, 10% glycerol, and 1% Triton X-100) plus protease inhibitors and 10 mM PR619 (inhibitor of ubiquitin isopeptidases). The cell lysates were rotated at 4 °C for 20 min and then clarified at 4 °C for 30 min at 14,000 × *g*. The proteins were immunoprecipitated using 20 ml of α-FLAG M2 beads with rotation overnight at 4 °C. For the 0 min sample, 5 mg/ml of FLAG-TNFα was added post-lysis. The beads were washed four times in lysis buffer and the samples were eluted by boiling in 60 µl of 1x SDS loading dye.

### Immunoprecipitation assay

For TNFR1 immunoprecipitation (IP), 1 × 10^7^ U937 cells were preincubated for 30 min with 1 mM Phenhydan^®^ or the appropriate amount of DMSO as a vehicle control. Next, the cells were treated for 0, 5, 15, 30, and 60 min, respectively, at 37 °C with 100 ng/ml FLAG-tagged or Fc-tagged human TNFα. The cells were collected into tubes pre-cooled on ice containing ice-cold PBS, centrifuged at 1,500 RPM, washed with ice-cold PBS, lysed in IP buffer (50 mM Tris-HCl [pH 7.4], 150 mM NaCl, 1% NP-40, 0.25%, Na-deoxycholate, 1% Triton X-100, 1 mM EDTA, 100 µM Na_3_VO_4_, and 1 mM C_7_H_7_FO_2_S (PMSF) and kept on ice for 20 min. For the 0 min sample, 100 ng/ml TNFα was added post-lysis. The cell lysates were clarified at 4 °C for 10 min at 14,000 × *g*. For IP, the lysates were incubated with 50 µl of µMACS™ Protein G MicroBeads (Miltenyi Biotec GmbH, Bergisch Gladbach, Germany) and rotated at 4 °C for 2 h. The lysates were applied to µ Columns (Miltenyi Biotec GmbH) pre-wetted with IP buffer, washed with IP buffer, and eluted with 40 µl of pre-warmed 95 °C SDS loading buffer. Of each sample (eluate), 20 µl was loaded for western blotting analysis.

### Acid Sphingomyelinase activity

Acid Sphingomyelinase (ASMase) activity was determined using an Acidic Sphingomyelinase Assay Kit from Abcam (Berlin, Germany) following the manufactures instructions. As indicated, U937 were pretreated at 37 °C for 30 min with vehicle or 1 mM Phenhydan^®^ followed by a stimulation with 100 ng/ml human TNFα for 30 min at 37 °C. Then, cells were lysed in ice-cold 10 mM Tris-HCl (pH 7.5), 50 mM NaCl, 1% Triton X-100, 30 mM Na_4_P_2_O_7_, 50 mM NaF, 100 μM Na_3_VO_4_, 2 μM ZnCl_2_, and 1 mM C_7_H_7_FO_2_S (PMSF) (modified Frackelton buffer). Cellular debris was removed by centrifugation (14,000 × *g*, 10 min, 4 °C). Lysate of 1 × 10^6^ cells each were used to determine ASMase activity by measuring of the fluorescence (λ_ex_ 540 nm and λ_em_ 590 nm) in the Infinite^®^ 200 PRO plate reader from Tecan (Männedorf, Switzerland).

### Sucrose gradient

We incubated 1 × 10^8^ U937 cells in 3 ml of RPMI 1640 medium at 37 °C for 30 min with 1 mM Phenhydan^®^, 10 mM MβCD, or vehicle alone (control). Subsequently, the cells were collected by centrifugation at 1,500 RPM, washed with ice-cold PBS, and incubated with 2 ml TX-buffer (25 mM Tris [pH 6.5], 150 mM NaCl, 5 mM EDTA, 1% Triton X-100 including protease inhibitor cocktail) at 4 °C for 3 h followed by sonication for 15 sec. The cell lysate was mixed with an equal volume of 95% sucrose (w/v) in T-buffer (25 mM Tris [pH 6.5], 150 mM NaCl, 5 mM EDTA) and placed into ultracentrifugation tubes. The cell lysate mixtures were overlaid sequentially with 4 ml of 35% (w/v) sucrose and 4 ml of 5% (w/v) sucrose, each in T-buffer, and centrifuged using an SW 32 Ti rotor (Beckman Coulter GmbH) for 19 h at 143,000 × *g*. We collected 1 ml of the fractions (numbered according to Fig. 3g from 1 to 7) from the top of the gradient and used them for protein analysis via western blotting.

### ImageStream analysis

For quantification of TNFR1 internalization by imaging flow cytometry, cells were incubated for 15 min at room temperature with vehicle or 1 mM Phenhydan^®^, respectively, followed by sedimentation and cooling on ice for 15 min. Labeling of TNFR1 with 100 ng/ml biotinylated TNFα and 5 µg/ml Alexa Fluor™ 488-conjugated Streptavidin was performed on ice for 20 min. To facilitate receptor internalization, the temperature was raised to 37 °C with pre-warmed medium for 30 min. Thereafter, the cells were fixed in 2% paraformaldehyde/PBS for 20 min without permeabilization. CellMask™ Deep Red plasma membrane stain (1:10,000 dilution of the stain) was added for the final 5 min. The cells were washed twice with PBS and the cell pellet was resuspended in 20 µl of PBS.

The ImageStream Mark II was used for cell analysis. At least 5000 images were acquired, detecting the internalization probes biotinylated TNFα/Alexa Fluor™ 488-conjugated Streptavidin (excitation: 488 nm) on Channel 2 and the cell surface label CellMask™ (excitation: 642 nm) on Channel 5. We applied the internalization wizard of the software Amnis IDEAS (version 6.0.154.0) according to the instruction manual, to determine the TNF/TNFR1 internalization rate in vehicle-treated versus Phenhydan^®^-treated U937 cells. For monitoring time-dependent CellMask™ fluorescence in the presence of Phenhydan^®^ 3 × 10^4^ U937 per value were resuspended in 300 µl PBS and pretreated with 1 mM Phenhydan^®^ or 10 mM methyl-β-cyclodextrin (MβCD) for 30 min, representing the time frame from −30 to 0 min. Following this, CellMask™ (1:10,000 dilution of the stain) was added in each well (0 up to 30 min). Conversely, cells were first incubated for 30 min with CellMask™ (−30 to 0 min) and then mixed with 1 mM Phenhydan^®^ (0 to 30 min). The measurements were performed at 37 °C in a black 96-well plate, and fluorescence (λ_ex_ 642 nm and λ_em_ 666 nm) was measured with the Infinite^®^ 200 PRO plate reader from Tecan. Samples were measured in triplicate every 15 min over a period of 1 h.

The fluorescence intensity of cholera toxin subunit B (CTxB) conjugated with Alexa Fluor™ 594 was also measured via flow cytometry using ImageStream Mark II (excitation: 590 nm on Channel 5). As indicated, U937 cells were pretreated at 37 °C for 30 min with vehicle or 1 mM Phenhydan^®^. Afterwards, the cells were washed with ice-cold PBS and stained with 1 µg/ml CTxB conjugated with Alexa Fluor™ 594 in PBS for 10 min at 4 °C in the dark. Then, the cells were washed three times with PBS, and an α-CTxB antibody (1:200) was added for 15 min at 4 °C in the dark to crosslink the CTxB in the lipid rafts. Subsequently, the cells were washed three times with ice-cold PBS and fixated with 4% paraformaldehyde for 10 min at room temperature in the dark, washed again and resuspended in PBS for the flow cytometer analysis.

### Statistical methods and analyses

For all experiments, the differences between datasets were considered statistically significant when *p*-values were <0.05, if not otherwise specified. Statistical comparisons were performed using the two-tailed Student’s *t* test. Asterisks are used in the figures to specify statistical significance (*p < 0.05; **p < 0.02; ***p < 0.001). The results are presented as means ± SD unless otherwise specified.

## Electronic supplementary material

Supplementary Table 1

Supplementary Figure 1

Supplementary Figure 2

Supplementary Figure 3

Supplementary Figure 4

Supplementary Figure 5

Supplementary Material
