# Supplementary figures and images for: The anticonvulsive Phenhydan® suppresses extrinsic cell death
Source: Cell Death Differ. 2018 Nov 15;26(9):1631–45. doi: 10.1038/s41418-018-0232-2 (PMC6748113; doi:10.1038/s41418-018-0232-2)

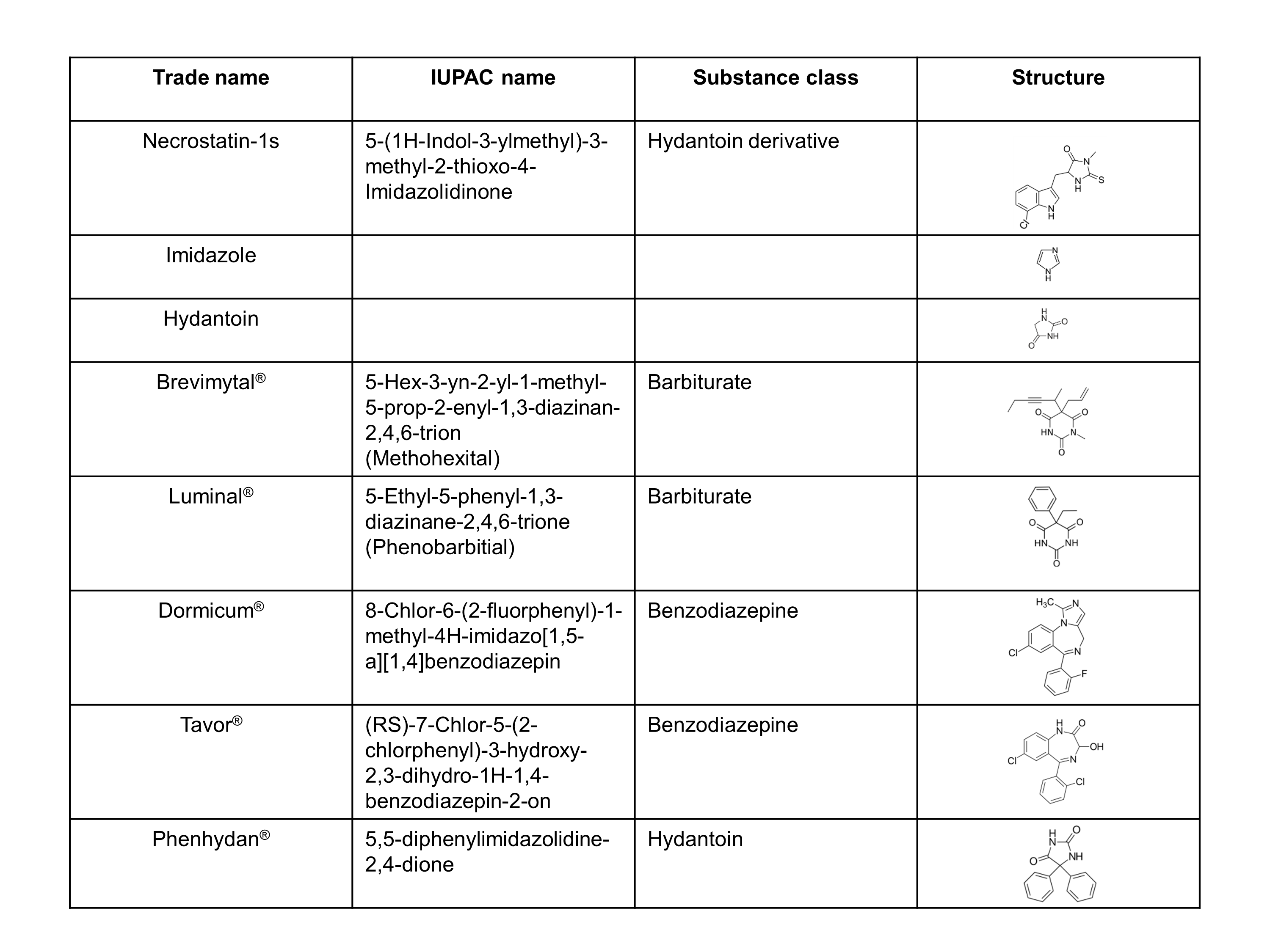

Supplement: Supplementary file 1 — Supplementary Table 1 [file 41418_2018_232_MOESM1_ESM.tif]

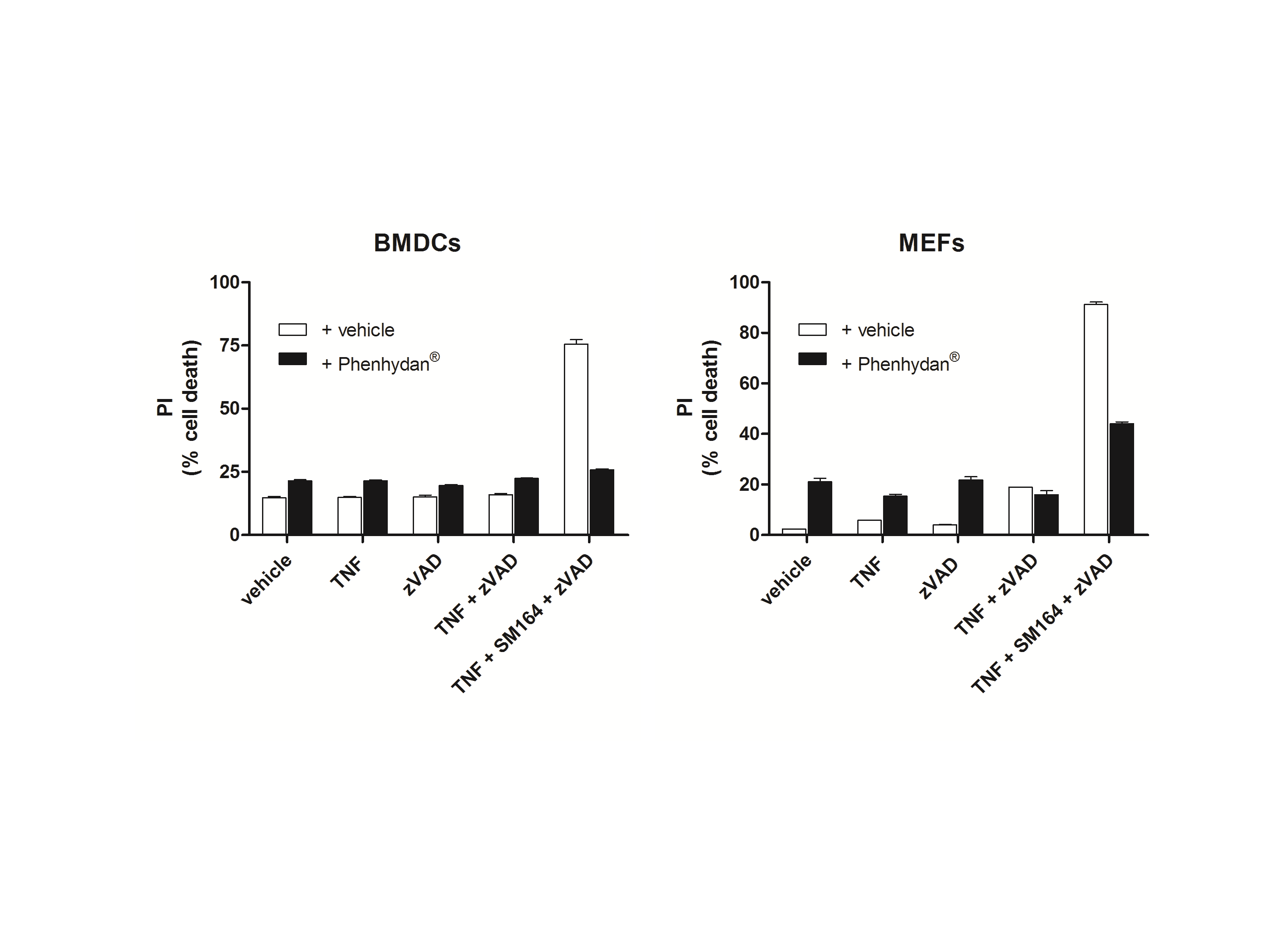

Supplement: Supplementary file 2 — Supplementary Figure 1 [file 41418_2018_232_MOESM2_ESM.tif]

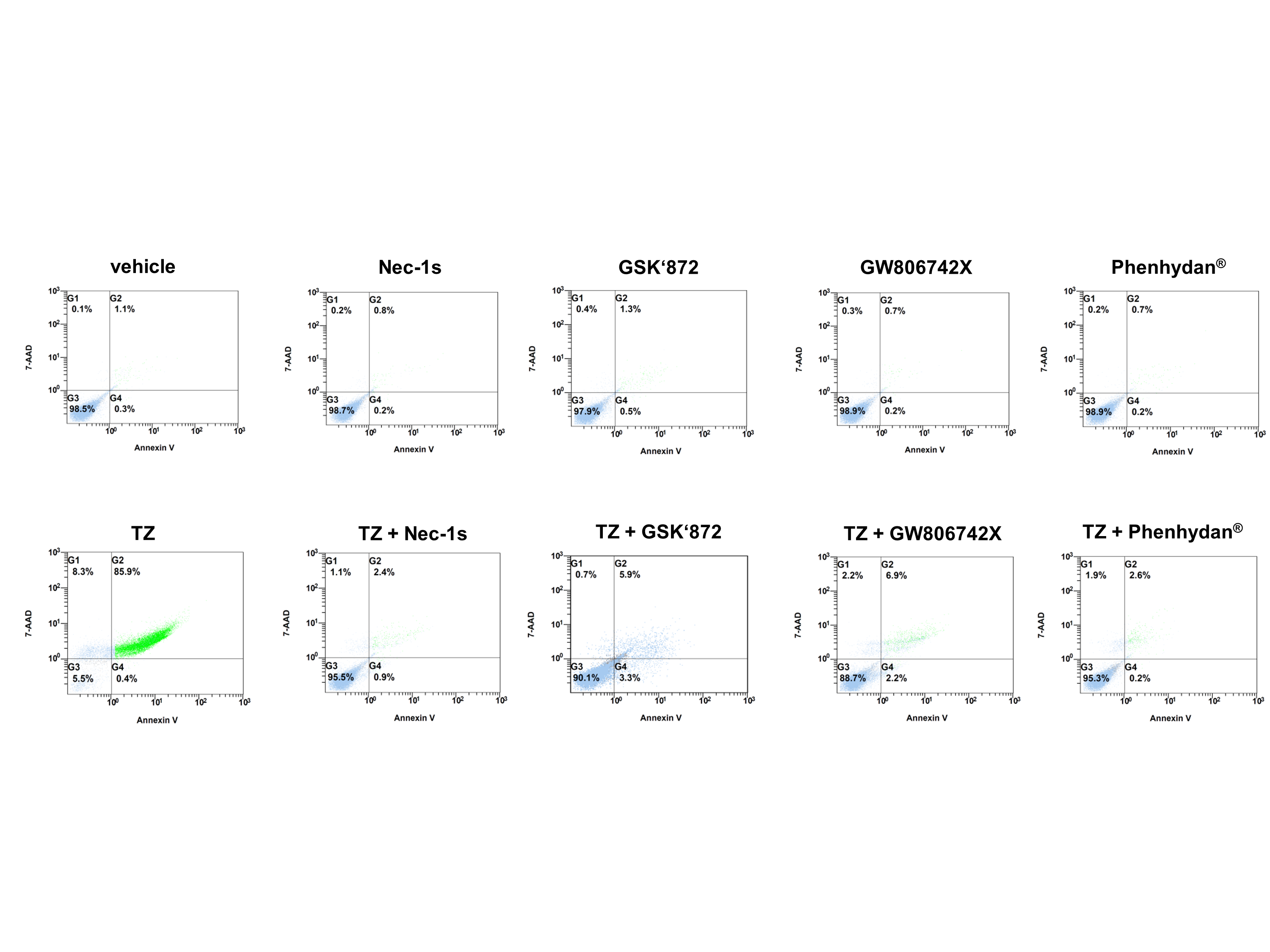

Supplement: Supplementary file 3 — Supplementary Figure 2 [file 41418_2018_232_MOESM3_ESM.tif]

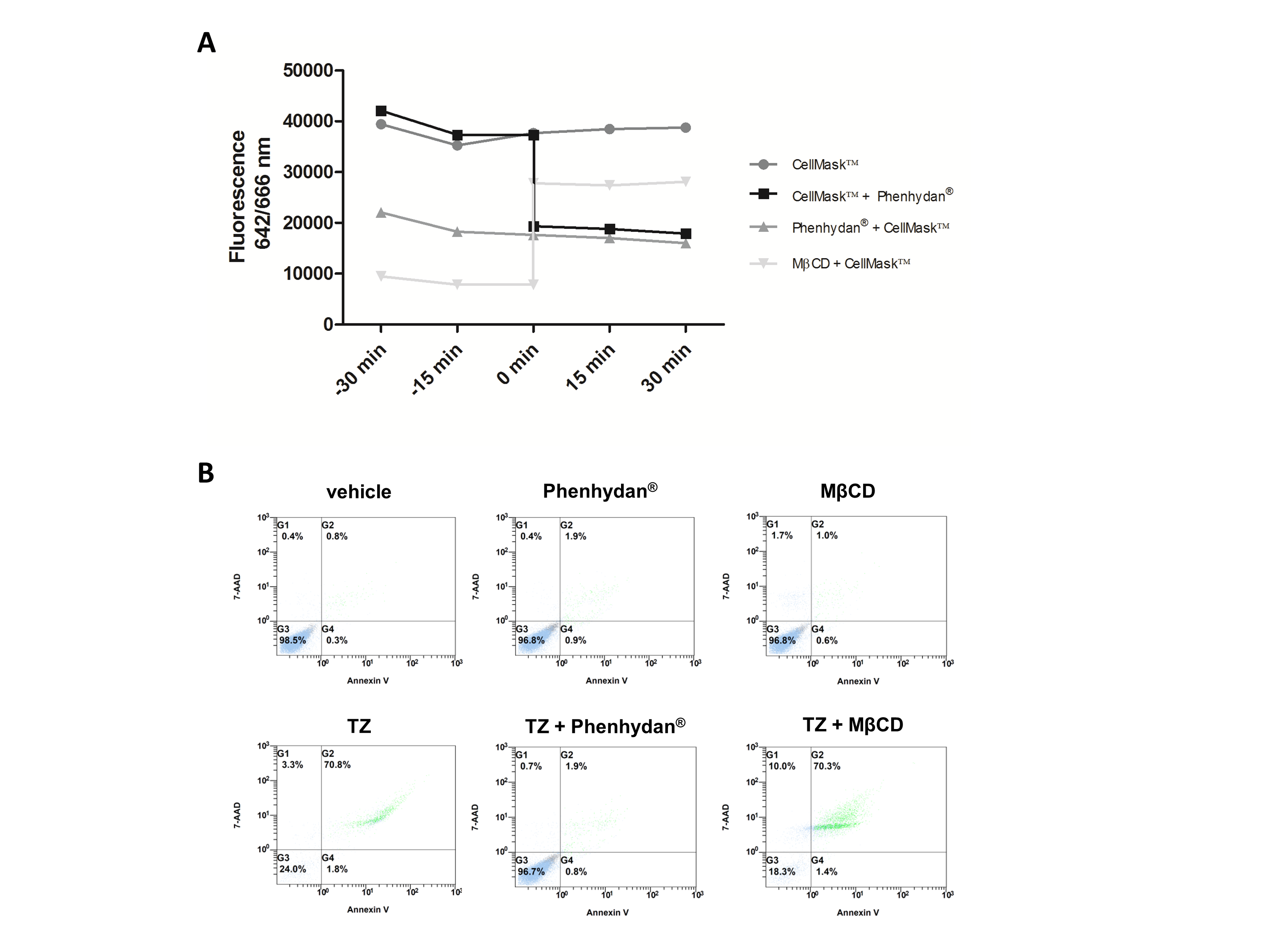

Supplement: Supplementary file 4 — Supplementary Figure 3 [file 41418_2018_232_MOESM4_ESM.tif]

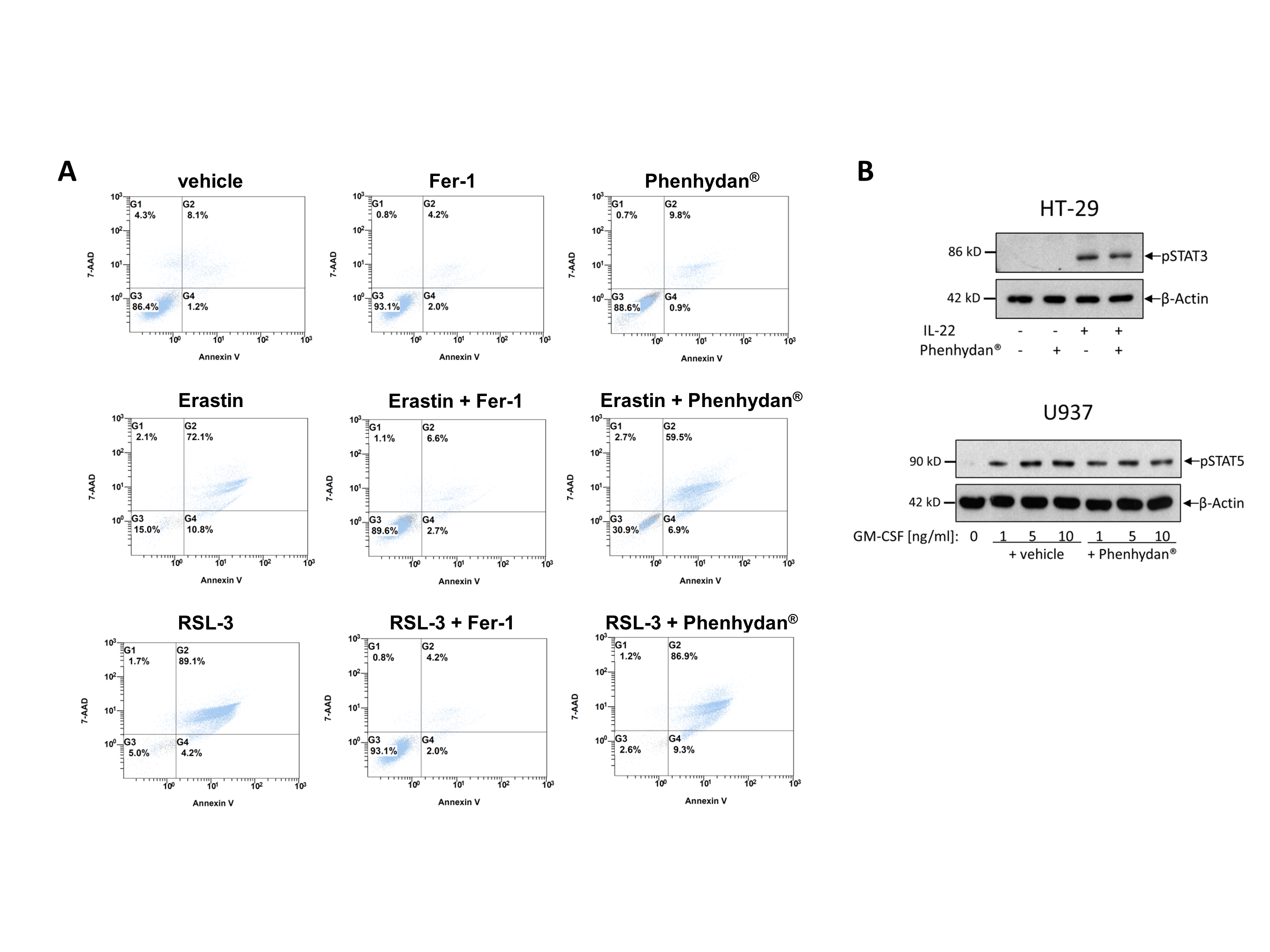

Supplement: Supplementary file 5 — Supplementary Figure 4 [file 41418_2018_232_MOESM5_ESM.tif]

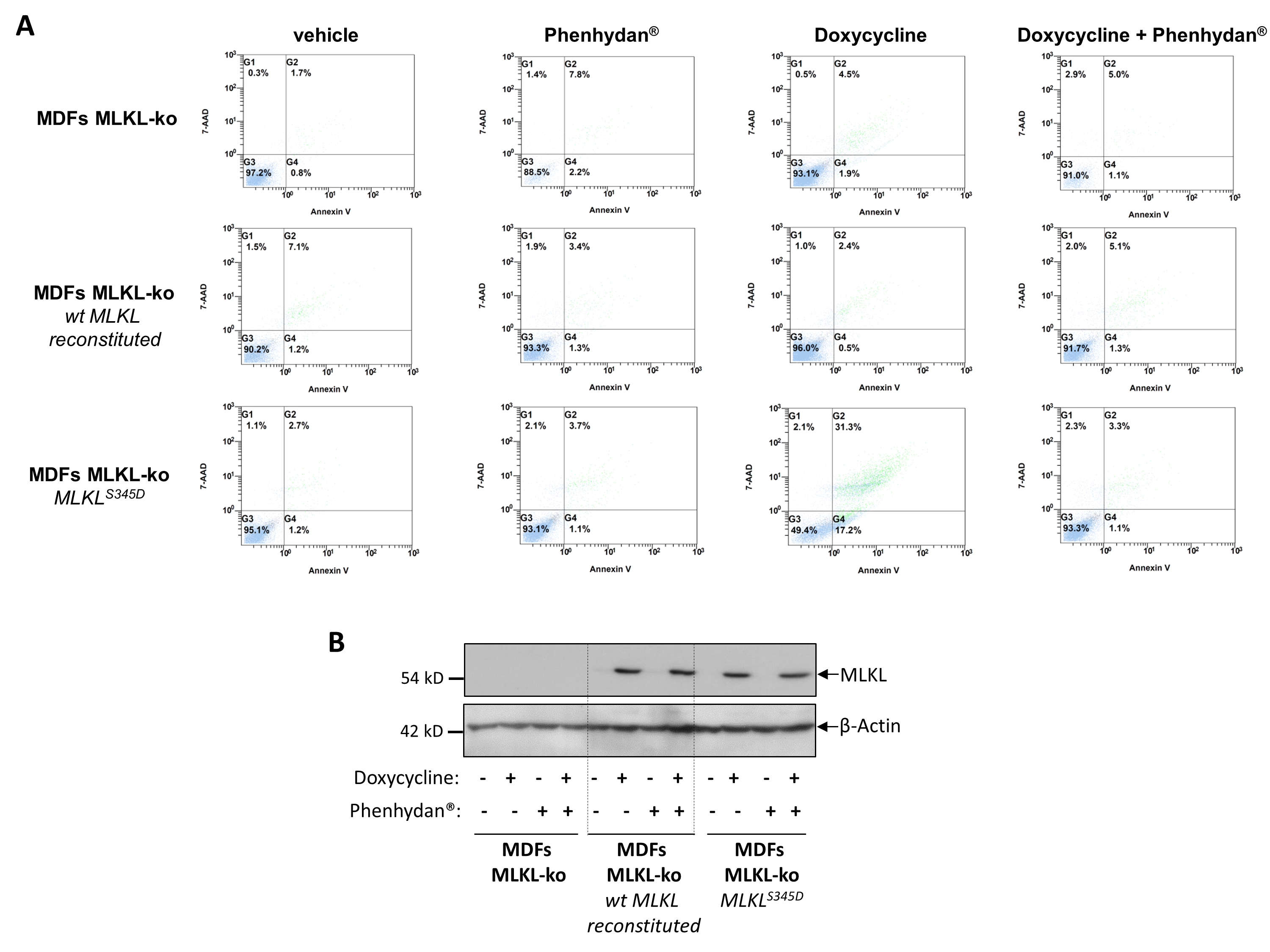

Supplement: Supplementary file 6 — Supplementary Figure 5 [file 41418_2018_232_MOESM6_ESM.tif]
