# Supplementary material for: The anticonvulsive Phenhydan® suppresses extrinsic cell death
Source: Cell Death Differ. 2018 Nov 15;26(9):1631–45. doi: 10.1038/s41418-018-0232-2 (PMC6748113; doi:10.1038/s41418-018-0232-2)
Supplement: Supplementary file 7 — Supplementary Material [file 41418_2018_232_MOESM7_ESM.docx]

**Supplementary Material**

**The anticonvulsive Phenhydan^®^ suppresses extrinsic cell death**

Caroline Moerke^1*^, Isabel Jaco^2*^, Christin Dewitz^1^, Tammo Müller^1^, Annette V. Jacobsen^3,4^, Jérémie Gautheron^5^, Jürgen Fritsch^6^, Jessica Schmitz^7^, Jan Hinrich Bräsen^7^, Claudia Günther^8^, James M. Murphy^3,4^, Ulrich Kunzendorf^1^, Pascal Meier^2^, and Stefan Krautwald^1^

^1^Department of Nephrology and Hypertension, University Hospital Schleswig-Holstein, 24105 Kiel, Germany

^2^Toby Robins Research Centre, Institute of Cancer Research, London SW3 6JB, UK

^3^The Walter and Eliza Hall Institute of Medical Research, Parkville, Victoria 3052, Australia

^4^Department of Medical Biology, University of Melbourne, Parkville, Victoria 3052, Australia

^5^Université Pierre et Marie Curie, UMR_S 938, Inserm, 75012 Paris, France

^6^Institute for Clinical Microbiology and Hygiene, University of Regensburg, 93053 Regensburg, Germany

^7^Department of Pathology, University of Hannover, 30625 Hannover, Germany

^8^Department of Medicine 1, Friedrich-Alexander-University, 91052 Erlangen, Germany

*C. Moerke and I. Jaco contributed equally to this paper

**Supplementary Figure Legends**

**Table 1**

Trade name, IUPAC name, substance class and chemical structure of all the substances used in the present study.

**Figure S1**

**Phenhydan^®^ suppresses RIPK1-mediated cell death also in primary cells.** (A) Primary bone marrow-derived macrophages and (B) MEFs were stimulated for 3 and 6 h, respectively, at 37°C with 100 ng/ml TNFα + 1 µM SMAC mimetic SM164 + 25 µM zVAD in the absence or presence of 1 mM Phenhydan^®^. As indicated, Phenhydan^®^ was added 30 min before induction of cell death. Necroptotic cell death was quantified by FACS analysis using PI as marker. Graphs show the mean ± SEM; *n* = 2-3 independent experiments.

**Figure S2**

**The protective effect of Phenhydan^®^ on TNF-induced cell death is as potent as that of commercially available but not FDA-approved inhibitors.** Murine L929 cells were stimulated at 37°C for 6 h with 100 ng/ml TNFα + 25 µM zVAD (TZ) in the absence or presence of different inhibitors of the necroptotic pathway. The cells were pretreated as indicated for 30 min with the commercially available inhibitors Nec-1s (50 µM), GSK’872 (1 µM), and GW806742X (2.5 µM), which were compared to 1 mM Phenhydan^®^. Necroptotic cell death was quantified by FACS analysis using 7-amino-actinomycin D and phosphatidylserine accessibility (Annexin V staining) as markers. Shown are data from one of three independent experiments.

**Figure S3**

**Phenhydan^®^ and MβCD do not inhibit the binding of the plasma membrane dye CellMask™ to the cell surface but merely Phenhydan^®^ inhibits TNF-mediated cell death.** (A) For monitoring the CellMask™ fluorescence spectrum of U937 cells in the presence and absence of the pharmaceuticals 3x 10^4^ cells per value were pretreated with 1 mM Phenhydan^®^ or 10 mM methyl-β-cyclodextrin (MβCD) for 30 min, representing the time frame from -30 to 0 min. Following this, CellMask™ (1:10,000 dilution of the stain) was added for the next 30 min. Conversely, cells were first incubated for 30 min with CellMask™ (-30 to 0 min) and then treated for 30 min with 1 mM Phenhydan^®^, representing the period from 0 to 30 min. The measurements were performed at 37°C in a black 96-well plate. Fluorescence (λ_ex_ 642 nm and λ_em_ 666 nm) was measured using the Infinite^®^ 200 PRO plate reader from Tecan. Samples were measured in triplicate every 15 min over a period of 1 h. (B) Murine NIH3T3 cells were stimulated at 37°C for 5 h with 100 ng/ml TNFα + 25 µM zVAD (TZ) in the absence or presence of 1 mM Phenhydan^®^ and 1 mM MβCD. Each of the pharmaceuticals was added 30 min before the induction of necroptosis. Necroptotic cell death was quantified by FACS analysis using 7-amino-actinomycin D and phosphatidylserine accessibility (Annexin V staining) as markers. The data are from one of three independent experiments**.**

**Figure S4**

**The inhibition of death receptor-mediated cell death by Phenhydan^®^ is specific.** (A) Phenhydan^®^ demonstrated no protective effect against erastin- or RSL3-induced ferroptosis. Murine NIH3T3 cells were stimulated at 37°C for 16 h with 10 µM erastin or 2 µM RSL3. 30-minute pretreatment of the cells with 1 µM ferrostatin-1 protected against cell death, illustrating that ferroptosis was the cell death mediator in this setting. In each case, 1 mM Phenhydan^®^ was added 30 min before the induction of ferroptosis. Ferroptotic cell death was quantified by FACS analysis using 7-amino-actinomycin D and phosphatidylserine accessibility (Annexin V staining) as markers. The data are from one of three independent experiments. (B) HT-29 cells were left untreated or were stimulated for 30 min with 100 ng/ml IL-22. Furthermore, U937 cells were left untreated or were stimulated for 60 min with 5 ng/ml GM-CSF. In each case, Phenhydan^®^ was added 30 min before stimulation with IL-22 and GM-CSF, respectively. Western blotting analysis of the cell lysates using the indicated phospho-STAT antibodies excluded a non-specific effect provoked by Phenhydan^®^ in the course of cytokine-mediated signal transduction.

**Figure S5**

**Doxycycline-induced expression of MLKL in wild-type and in mutated MDFs (MLKL^S345D^) is not affected by the presence of Phenhydan^®^.** (A) Mouse dermal fibroblasts (MDFs) isolated from MLKO-ko animals, MDFs from MLKL-ko animals in which wild-type (wt) MLKL was reconstituted, and the constitutively active MLKL mutant S345D were induced as indicated for 7 h at 37°C with 0.5 µg/ml doxycycline in the absence or presence of 1 mM Phenhydan^®^ (Phenhydan^®^ was introduced 30 min before the addition of doxycycline). Each probe was split for FACS analysis (A) and corresponding western blotting (B). Doxycycline-induced cell death was quantified by FACS analysis using 7-amino-actinomycin D and phosphatidylserine accessibility (Annexin V staining) as markers. The data are from one of three independent experiments. (B) Identical samples to those analyzed by FACS (A) were used for the detection of whole MLKL expression by western blotting, using an anti-MLKL antibody (clone 3H1). The blot was re-developed with an antibody against β-actin as the loading control.
